# Government and University Partner for Virtual Service-Learning to Support Intergenerational Social Interaction

**DOI:** 10.1093/geroni/igab046.1412

**Published:** 2021-12-17

**Authors:** Betsy Kemeny, Adelle Williams, Stephanie Cole

**Affiliations:** 1 Slippery Rock University, Slippery Rock, Pennsylvania, United States; 2 State of Pennsylvania, Harrisburg, Pennsylvania, United States

## Abstract

Pre-pandemic, evidence existed that intergenerational service-learning programs support knowledge of aging and positive attitudes and perceptions (Monahan et al., 2020). As spring 2020 COVID-19 lock downs and public health warnings urged physical distancing of community dwelling older adults, growing concern about the unintended consequences of increased social isolation on mental and physical health prompted the Secretary’s Office of Pennsylvania Department of Aging to design a pilot project with university faculty for virtual intergenerational social interaction. The Department identified older adults at the highest risk for social isolation (live alone, in poverty, with a disability). The resulting pilot project is fully integrated as a high impact practice into eight sections of recreational therapy and gerontology courses with participation by 210 undergraduate students and 210 older adults for 9 weeks of both the fall and spring semesters. Students, who received extensive classroom instruction aimed at avoiding negative stereotypes of older adults as helpless and dependent, called their assigned partner several times a week for at least an hour of communication. Using the UCLA loneliness scale, community-dwelling older adults reported frustration with isolation due to the pandemic. Those with low and moderate loneliness reported positive feelings about program and looking forward to interactions with students. Students gained virtual communication skills that may contribute to telehealth competencies, intervention skills such as assessment, life review/reminiscence, mindfulness techniques, and leisure education. Moreover, an analysis of student reflections revealed positive changes in attitudes toward older adults and the ability to enjoy common interests despite age differences.

